# Lovastatin in *Aspergillus terreus*: Fermented Rice Straw Extracts Interferes with Methane Production and Gene Expression in *Methanobrevibacter smithii*

**DOI:** 10.1155/2013/604721

**Published:** 2013-04-28

**Authors:** Mohammad Faseleh Jahromi, Juan Boo Liang, Yin Wan Ho, Rosfarizan Mohamad, Yong Meng Goh, Parisa Shokryazdan, James Chin

**Affiliations:** ^1^Institute of Tropical Agriculture, Universiti Putra Malaysia (UPM), 43400 Selangor, Serdang, Malaysia; ^2^Institute of Bioscience, Universiti Putra Malaysia (UPM), 43400 Selangor, Serdang, Malaysia; ^3^Faculty of Biotechnology and Biomolecular Sciences, Universiti Putra Malaysia (UPM), 43400 Selangor, Serdang, Malaysia; ^4^Institute of Tropical Forestry and Forest Products, Universiti Putra Malaysia (UPM), 43400 Selangor, Serdang, Malaysia; ^5^Faculty of Veterinary Medicine, Universiti Putra Malaysia (UPM), 43400 Selangor, Serdang, Malaysia; ^6^Faculty of Agriculture and Food Science, University of Queensland, Gatton, QLD 4343, Australia

## Abstract

Lovastatin, a natural byproduct of some fungi, is able to inhibit HMG-CoA (3-hydroxy-3methyl glutaryl CoA) reductase. This is a key enzyme involved in isoprenoid synthesis and essential for cell membrane formation in methanogenic Archaea. In this paper, experiments were designed to test the hypothesis that lovastatin secreted by *Aspergillus terreus* in fermented rice straw extracts (FRSE) can inhibit growth and CH_4_ production in *Methanobrevibacter smithii* (a test methanogen). By HPLC analysis, 75% of the total lovastatin in FRSE was in the active hydroxyacid form, and *in vitro* studies confirmed that this had a stronger effect in reducing both growth and CH_4_ production in *M. smithii* compared to commercial lovastatin. Transmission electron micrographs revealed distorted morphological divisions of lovastatin- and FRSE-treated *M. smithii* cells, supporting its role in blocking normal cell membrane synthesis. Real-time PCR confirmed that both commercial lovastatin and FRSE increased (*P* < 0.01) the expression of HMG-CoA reductase gene (*hmg*). In addition, expressions of other gene transcripts in *M. smithii*. with a key involvement in methanogenesis were also affected. Experimental confirmation that CH_4_ production is inhibited by lovastatin in *A. terreus*-fermented rice straw paves the way for its evaluation as a feed additive for mitigating CH_4_ production in ruminants.

## 1. Introduction

**Figure 1:**
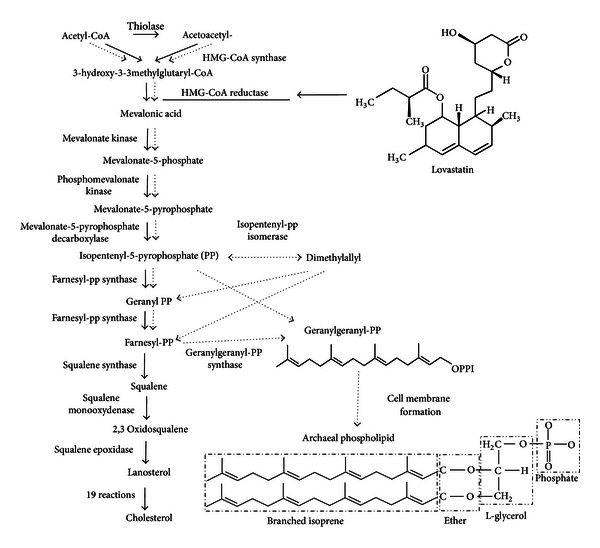
Biosynthesis pathway of cholesterol production in humans (solid-line markers) and phospholipids production in Archaea (dotted-line markers). HMG-CoA reductase is the common enzyme converting HMG-CoA to mevalonic acid in the two pathways. Lovastatin is an inhibitor of HMG-CoA reductase and thus reduces the production of mevalonic acid in both pathways (modified from http://ourbiochemistry.blogspot.com/2008/08/29-cholesterol-synthesis.html).

**Figure 2:**
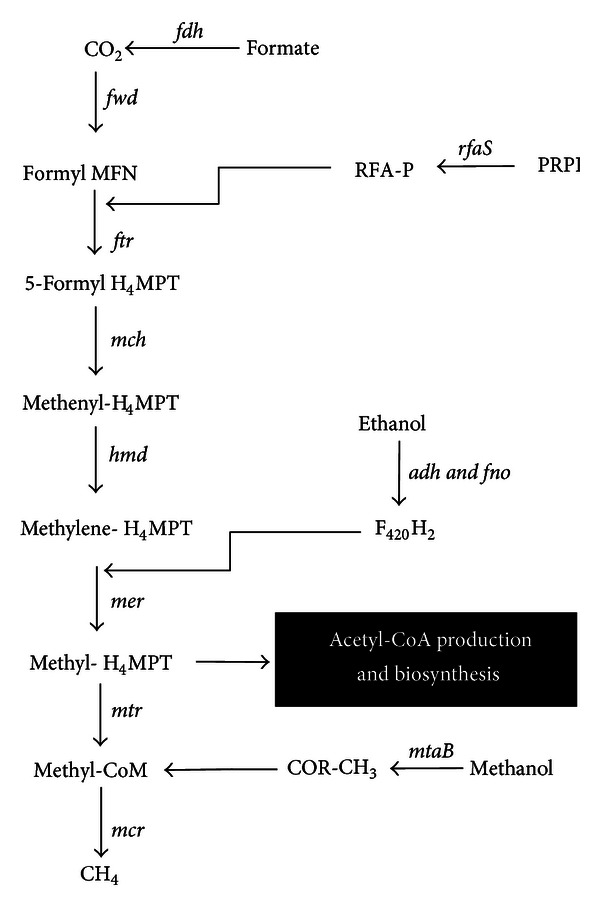
Methanogenesis pathway in *M. smithii*. *M. smithii* is able to use the Methyl H_4_MPT in the middle step of methanogenesis pathway for production of acetyl-CoA and biosynthesis reactions. Enzymes and genes encode: formate dehydrogenase (*fdh*); formyl-MF dehydrogenase (*fwd*); MFN, methanofuran; ribofuranosylaminobenzene 5_-phosphate (RFA-P); ribofuranosylaminobenzene 5_-phosphate synthase (*rfaS*); 5-phospho-a-D-ribosyl-1-pyrophosphate (PRPP); formyl-MF: H_4_MPT formyltransferase (*ftr*); tetrahydromethanopterin (H_4_MPT); methenyl-H_4_MPT cyclohydrolase (*mch*); methylene-H_4_MPT dehydrogenase (*hmd*); methylene-H_4_MPT reductase (mer); methyl-H_4_MPT: HS-CoM methyltransferase (*mtr*); alcohol dehydrogenase (Adh); F420-dependent NADP oxidoreductase (*fno*); methanol : cobalamin methyltransferase (*mtaB*); methyl CoM Reductase (*mcr*) (modified from Hendrickson et al. [14]).

The formation of isoprenoid chains is a key component of membrane phospholipid synthesis in Archaea. This pathway requires the production of mevalonic acid from 3-hydroxy-3methyl glutaryl CoA catalyzed by the enzyme HMG-CoA reductase, a critical rate-limiting step shared in common with cholesterol biosynthesis in humans (Figure 1). Lovastatin is a natural polyketide synthesized by *Aspergillus terreus* and *Pleurotus ostreatus *(oyster mushroom), where it may occur at concentrations as high as 2.8% dry weight [1]. Lovastatin prescribed at a dosage of 80 mg daily can dramatically reduce cholesterol levels by 40% simply through the inhibition of HMG-CoA reductase activity. Through interference with membrane synthesis (Figure 1), lovastatin can inhibit the growth of methanogenic Archaea in the rumen without adverse effects on other cellulolytic bacteria [2] and, in this way, mediates reduction in methane (CH_4_) release into the environment. However, the high cost of lovastatin preempts its use as a feed additive in the mitigation of ruminal CH_4_ production. Another approach that may be economically viable is to incorporate *A. terreus* as a feed supplement and inhibitor of methanogenic Archaea that produces methane in the process of methanogenesis (Figure 2). Furthermore, since *A. terreus* is a known producer of cellulolytic enzymes [3–5], it complements the degradation of lignocellulose components in the rumen enhance feed conversion efficiency. This paper describes a series of experiments to test the hypothesis that lovastatin generated by *A. terreus* fermentation of rice straw (fungal treated rice straw extracts or FRSE) inhibits the growth and methanogenesis by *Methanobrevibacter smithii* (DSM 861), a gastrointestinal methanogen similar to the dominant species in the rumen. The molecular mechanism for this effect was also elucidated by real-time PCR. 

## 2. Materials and Methods

### 2.1. Substrate, Microorganism, and Spore Suspension

Rice straw (RS) was collected from the local rice fields in the state of Selangor, Malaysia. The material was dried and ground to uniform size (No. 6 mesh) and stored in plastic bags at 4°C for later use as a substrate. 

*A. terreus *ATCC 74135, obtained from American Type Culture Collection (ATCC), was maintained on potato dextrose agar (PDA) slants at 25°C for 7 days, stored at 4°C, and subcultured every two weeks. Spore suspension was prepared in 0.1% Tween-80 solution in approximately 10^7^ spores/mL concentration.

### 2.2. Solid-State Fermentation

Solid state fermentation of RS was carried out in 2 L Erlenmeyer flasks. About 200 grams of RS, 200 mL distilled water (containing 1% urea) were added to give moisture content of approximately 50%. The flasks were plugged with cotton-wool and autoclaved at 121°C for 15 min prior to inoculate with 40 mL of an *A. terreus* spore suspension (containing 10^7^ spores/mL). A sample was fermented at 25°C for 8 days, conditions which had previously been found to be optimal [6]. At the end of fermentation, the sample was dried at 60°C for 48 h. 

### 2.3. Preparation of FRSE 

For preparation of FRSE, 200 g of the fermented rice straw was mixed with 1.5 L of methanol and shaken for 2 h at room temperature. The solid samples were removed from the suspension by vacuum filtration (0.45 *μ*m pore size, Pall Corporation, Ann Arbor, MI). Methanol was removed by rotary evaporation at 45°C (Eppendorf, USA), and the solid residual or FRSE was used in the further experiments. 

### 2.4. Lovastatin Quantification by HPLC

The concentration of lovastatin in the FRSE was quantified using HPLC (Waters, USA, 2690) and an ODS column of Agilent (250 × 4.6 mm i.d., 5 *μ*m). The mobile phase consisted of acetonitrile and water (70 : 30 by volume) containing 0.5% acetic acid. The UV photo diode array (PDA) detection range was set from 210 to 400 nm, and lovastatin was detected at 237 nm. The sample injection volume was 20 *μ*L, and the running time was 15 min. Different concentrations of lovastatin (mevinolin, 98%, HPLC grade, sigma, M2147) were used as standard.

### 2.5. Microorganism and Anaerobic Microbial Culture

*Methanobrevibacter smithii *DSM 861 used in this study was obtained from the German Resource Centre for Biological Material (DSMZ, Germany). The Balch medium 1 was used for the growth of *M. smithii* with some modification on it containing 0.45 g/L of K_2_HPO_4_, 0.45 g/L of KH_2_PO_4_, 0.45 g/L of (NH_4_)_2_SO_4_, 0.9 g/L of NaCl, 0.12 g/L of CaCl_2_·2H_2_O, 0.19 g/L of MgSO_4_·7H_2_O, 2.5 g/L of NaHCO_3_, 2.0 g/L of Trypticase, 2.0 g/L of yeast extract, 2.5 g/L of sodium acetate, 2.5 g/L of sodium formate, 4.9 × 10^−5^ g/L of coenzyme M (sodium 2-mercaptoethane-sulfonate), 0.5 g/L of cysteine·HCl, 0.5 g/L of Na_2_S·9H_2_O, and 0.001 g/L resazurin (pH 6.9). Vitamin and trace mineral solutions were added according to Balch et al. [7], and a VFA mixture was added according to Lovley et al. [8]. The mixture was flushed with CO_2_, and approximately 10 mL of medium was transferred into 50 mL serum bottles under anaerobic conditions. The bottles were closed by rubber stoppers and aluminum seals and autoclaved in 121°C for 15 min. Lovastatin in final concentrations of 1, 10, and 50 *μ*g/mL and FRSE in final concentration of 10, 100, and 500 *μ*g/mL were filter-sterilized using 0.2 *μ*m sterile syringe filters (Pall/Gelman, East Hills, NY, USA) and added into the medium after autoclaving. The samples were inoculated with 5% of a 72 h culture of *M. smithii*. The gas phase in each bottle was exchanged with an 80% H_2_-20% CO_2_ gas mixture at 100 kPa. The bottles were incubated at 39°C for 72 h. Growth was monitored from the optical density at 620 nm. 

### 2.6. Methane Determination

The concentration of CH_4_ in the headspace gas phase was determined with an Agilent 6890 Series Gas Chromatograph (Wilmington, DE, USA). Separation of the gases was achieved using an HP-Plot Q column (30 m × 0.53 mm × 40 *μ*m) (Agilent Technologies, Wilmington, DE, USA) with N_2_ as the carrier gas with a flow rate of 3.5 mL/min (MOX, Kuala Lumpur, Malaysia). The isothermal oven temperature was 50°C, and separated gases were detected using a thermal conductivity detector. Methane was eluted in 4 min. Calibration used standard gas prepared by Scott Specialty Gases (Supelco, Bellefonte, PA, USA), which contain 1% of CH_4_, CO, CO_2_, O_2_, and H_2_ in N_2_.

### 2.7. RNA Extraction and Gene Expression 

Cells from two milliliters of culture were harvested by centrifugation at 10,000 rpm for 2 min at 4°C and directly used for RNA extraction. RNA was extracted using the RiboPure Bacteria RNA Isolation kit (AMBION, AM1925, Austin, TX, USA) according to the manufacturer's protocol and reverse transcribed into cDNA using First Strand cDNA synthesis Kit according to the manufacturer's instructions (Maxime RT-PCR Kit, iNtRON, Germany). In the next step, Real-time PCR was performed with the BioRad CFX96 Touch (Bio-Rad, USA) using optical grade plates. The PCR reaction was performed on a total volume of 25 *μ*L using the iQSYBR Green Supermix (BioRad, USA). Each reaction included 12.5 *μ*L SYBR Green Supermix, 1 *μ*L of each Primer, 1 *μ*L of cDNA samples, and 9.5 *μ*L H_2_O. All real-time PCRs were performed in duplicate. Primers used in this study are shown in Table 1. 16S rRNA was used as reference gene [9]. The 2^−ΔΔCT^ method was used for determination of relative gene expression [10]. Results of the real-time PCR data were represented as CT values of the threshold cycle number at which amplified product was first detected. ΔCT is difference in CT value of the target gene from the CT value of the reference gene (16S rRNA). ΔΔCT is ΔCT of treatment samples (lovastatin and FRSE) minus ΔCT of the untreated control. Data is presented as fold change expression in the target gene of a treatment sample compared to the normal sample. 

### 2.8. Transmission Electron Microscopy (TEM)

The procedure of sample preparation of Hayat [11] with minor modified [12] by the Electron Microscopy Unit, Institute of Bioscience, Universiti Putra, Malaysia, was used for the TEM study. A Hitachi H-7100 (Japan) transmission electron microscope was used.

### 2.9. Statistical Analysis

All of the experiments were performed in triplicate. Data were analyzed as a completely randomized design (CRD) using the general linear model (GLM) procedure of SAS 9.2 [13]. All multiple comparisons among means were performed using Duncan's new multiple range test (*α* = 0.05).

## 3. Results

### 3.1. Purity of Lovastatin

**Figure 3:**
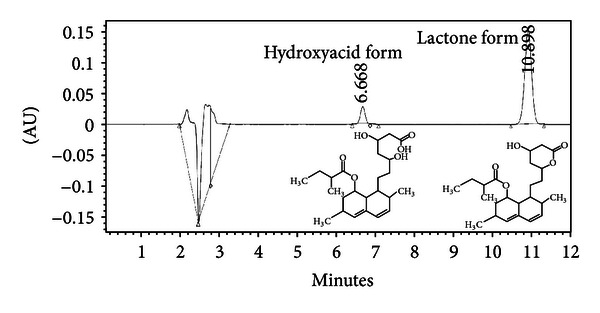
Molecular structure and HPLC chromatogram of lovastatin.

The purity of lovastatin in FRSE was compared with the commercially available form by HPLC.  Figure 3 shows that commercial lovastatin was >98% in the lactone form. In contrast, the yield of lovastatin was 97 mg/g dry matter in FRSE, and approximately 75% of this was in the bioactive hydroxyacid form (73 mg/g DM). More information about production of lovastatin by *A. terreus* in solid state fermentation was published in our previous paper [6]. To evaluate the effectiveness of the commercial and FRES lovastatin, 3 dilutions representing 1, 10, and 50 *μ*g/mL of commercial lovastatin and 10, 100, and 500 *μ*g/mL of FRSE (contain lovastatin) were used to investigate the biological activity of lovastatin on growth morphology, methane production, and gene transcript activity.

### 3.2. Microbial Growth and CH_4_ Production

**Figure 4:**
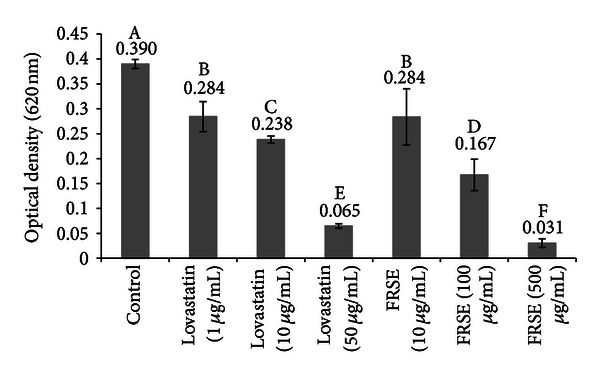
The effect of the addition of commercial lovastatin and fermented rice straw extract (FRSE) in the broth culture of *M. smithi* on growth rate after 72 h of incubation. The error bar represents one standard deviation. A, B, C, D, E and F indicating differences between means (*P* < 0.01).

Treatment with commercial and FRSE lovastatin significantly (*P* < 0.01) inhibited the growth of *M. smithii *(Figure 4). Inhibition by commercial lovastatin at 10 and 50 *μ*g/mL was similar to that of 100 and 500 *μ*g/mL FRSE, respectively. At the same concentration of total lovastatin, the growth inhibitory effect of FRSE on *M. smithi* was much stronger than when commercial lovastatin was used alone.

**Figure 5:**
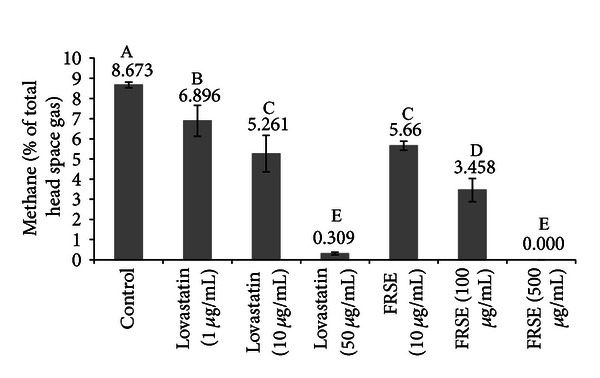
The effect of the addition of commercial lovastatin and fermented rice straw extract (FRSE) in the broth culture of *M. smithi* on methane production (as % of total headspace gas) after 72 h of incubation. The error bar represents one standard deviation. A, B, C, D, and E indicate differences between means (*P* < 0.01).

Commercial lovastatin and FRSE also inhibited CH_4_ production after 72 h of incubation (Figure 5). At the same concentration of total lovastatin, CH_4_ production in the FRSE treatments was lower than treatments containing commercial lovastatin. There was no significant difference in CH_4_ production by 10 *μ*g/mL commercial lovastatin and 10 *μ*g/mL FRSE (equivalent to 1 *μ*g/mL total lovastatin) while CH_4_ was not detected in cultures containing 500 *μ*g/mL FRSE (Figure 5). 

### 3.3. Microbial Morphology

**Figure 6:**
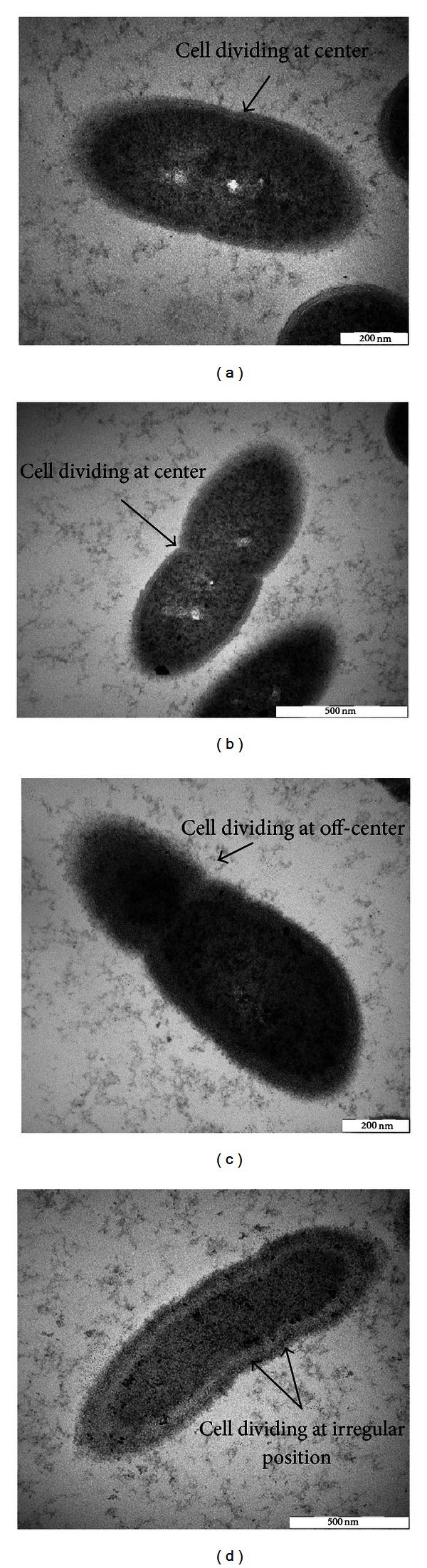
Transmission electron micrograph of *M. smithii* as affected by commercial lovastatin (c) and fermented rice straw extract (FRSE) (d). Division of normal *M. smithii* cells (a) and (b) occurred at the middle forming two equal cells. In cultures treated with commercial lovastatin (c) and FRSE (d), cell division was off-center and irregular. Pictures were obtained using a Hitachi H-7100 (Japan) transmission electron microscope (TEM).

Following growth with commercial lovastatin or FRSE, the morphology of *M. smithii* was greatly altered (Figure 6). The lines of cell division in *M. smithii* for the control samples (Figures 6(a) and 6(b)) displayed symmetrical cell division, while mitotic figures in treated samples were off-centered resulting in aberrant division figures. 

### 3.4. Gene Expression

**Figure 7:**
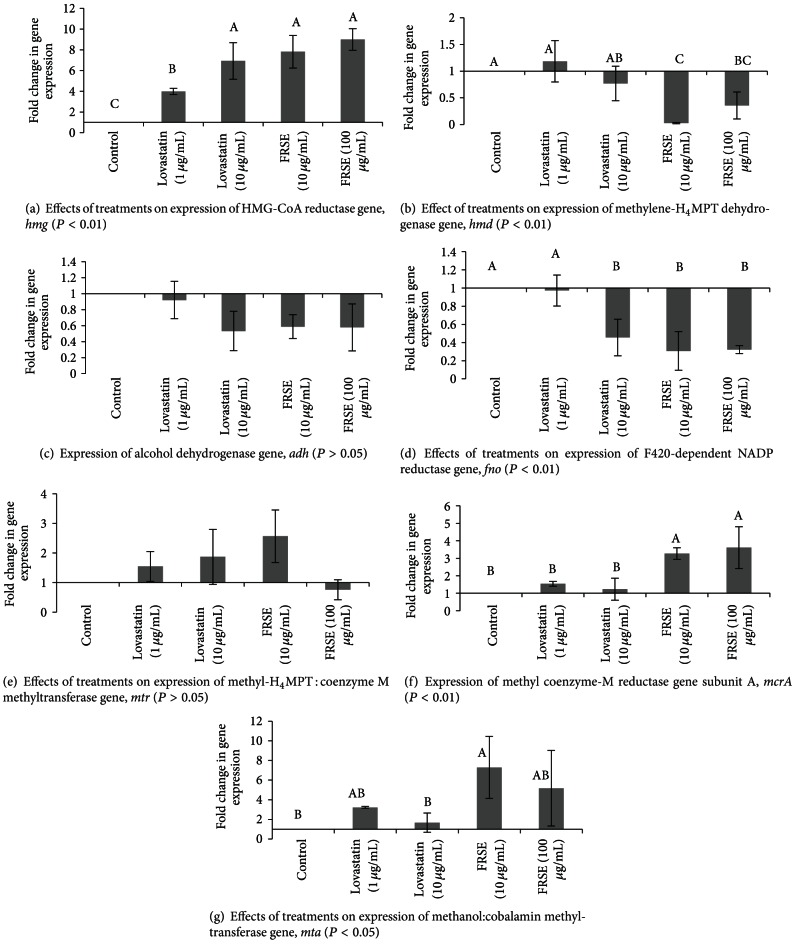
Effect of lovastatin and FRSE on genes expression in *M. smithii. *16S rRNA was used as reference gene. Data were normalized by control and reference genes. Error bar represent standard deviation. Letters on the columns indicating differences between means (*P* < 0.05).

To obtain some insight into the mechanism of decreased CH_4_ production in *M. smithii, *real-time PCR was used to analyse the effect of commercial lovastatin and FRSE on expression of some of the key genes involved in the methanogenic pathway (Figure 2). Since little growth and CH_4_ are produced by *M. smithii* in the high concentration of lovastatin and FRSE and it is not possible to extract sufficient quantity of RNA in these samples, RNA was extracted only from control and two lower levels of treatments. Both commercial lovastatin and FRSE significantly increased the expression of HMG-CoA reductase gene (*hmg*) (*P* < 0.05). Fold change in expression of this gene in FRSE treated cells was higher than those treated with commercial lovastatin (Figure 7(a)), and the maximal change caused by the 50 *μ*g/mL FRSE was a 9-fold increase.

The FRSE treatments, but not commercial lovastatin, also had a significant effect on expression of methylene-H_4_MPT dehydrogenase gene (*hmd*) that encodes the enzyme for conversion the methenyl-H_4_MPT into methylene-H_4_MPT (Figure 2). Similarly, with commercial lovastatin and FRSE reduced the expression of alcohol dehydrogenase gene (*adh*), this reduction was not significant (*P* > 0.05) (Figure 7(c)). Treatments containing 10 *μ*g/mL Lovastatin, 10 *μ*g/mL and 50 *μ*g/mL FRSE significantly (*P* < 0.01) reduced the expression of F420-dependent NADP reductase gene (*fno*) in *M. smithii* (Figure 7(d)). L-lovastatin and FRSE increased the expression of methyl-H_4_MPT : coenzyme M methyltransferase gene (*mtr*) (Figure 7(e)). This gene produces the enzyme for the transfer of methyl group from methyl-H_4_MPT to HS-COM [15]. Methyl coenzyme-M reductase (*mcr*) is the last enzyme in the methanogenesis pathway. The effect of lovastatin and FRSE on expression of this gene is shown in Figure 7(f). The result shows that lovastatin has no effect on the expression of this gene, but FRSE at both levels increased the expression of this gene in *M. smithii* (*P* < 0.01). Methanol : cobalamin methyltransferase gene (*mta*) is the gene for encoding of methanol : cobalamin methyltransferase that catalyses the conversion of methanol into COR-CH_3_ and production of CH_4_ in the process of methanogenesis. Both lovastatin and FRSE significantly increase the expression of this gene in *M. smithii* (*P* < 0.01) (Figure 7(g)). The enhancement effect of FRSE on expression of this gene was higher than L-lovastatin.

## 4. Discussion

Lovastatin is an effective therapy in the treatment of hypercholesterolemia because of its ability to inhibit HMG-CoA reductase activity, a key enzyme involved in cholesterol synthesis [16]. Because of this, it is easy to ignore the fact that generic fungal statins have evolved to allow producer strains to gain a competitive survival advantage in complex ecological communities by interfering with the assembly of isoprenoid chains required for membrane phospholipid synthesis [17]. In this way, statin-producing fungi can arrest the growth rates of susceptible strains [18] by interfering with cell wall formation and arresting cellular division [19]. To test whether such a strategy could be used for the reduction of methane production by ruminants, it was necessary to show firstly that rice straws fermented with a representative statin-producing fungal strain of *Aspergillus terreus*, was capable of synthesizing biologically active lovastatin. HPLC confirmed that while commercial lovastatin existed primarily in a biologically inactive lactone (L) form, the biologically active hydroxyacid or H-form predominated in FRSE (Figure 3).

 In the second stage, it was necessary to demonstrate that the lovastatin in FRSE was able to exert a biological impact on growth and cell membrane assembly in the target experimental methanogen—*M. smithii. *As shown in Figure 4, growth rates of *M. smithii* were inhibited by both commercial lovastatin and FRSE. At the same time, electron micrographs of *M. smithii *showed abnormal formation of cell membranes in mitosis, presumably caused by interference in the synthesis of isoprenoid building blocks. Although both treatments significantly (*P* < 0.01) inhibited the growth of *M. smithii, *the growth inhibitory effect of FRSE on *M. smithi* was much stronger than when control L-lovastatin was used alone. It is likely that this could have been the consequence of having to convert the lactone form to the hydroxy form of lovastatin before the inhibition of HMG-CoA reductase can occur in *M. smithii*.

Commercial lovastatin contains 2% of the active H-form of lovastatin, so their titrations of 1, 10, and 50 ug/mL represent 0.02, 0.20, and 1 ug H-form per mL. The FRSE contains 7.3% H-form of lovastatin and 10, 100, and 500 ug DM/mL FRSE are actually 0.73, 7.3, and 36.5 ug/mL H-lovastatin. Thus, their lowest concentration of FRSE is similar in H-form content with their highest commercial lovastatin concentration, which was much more inhibitory in the experiments shown in both Figures 4 and 5. Similarly, in Figure 4, 1 ug/mL of the commercial form appears to be roughly equivalent to 10 ug/mL of FRSE. In Figure 5, 10 ug/mL of the commercial form appears to be roughly equivalent to 10 ug/mL of FRSE. This would seem to suggest that on a total (H + L-form) lovastatin basis, the FRSE appears to be roughly equivalent to possibly 10-fold more active. 

 It is important to note that this activity of lovastatin against a methanogen operates differently from its antiproliferative activity against eukaryote cells. Damage of the human cell division by lovastatin has been reported in a previous study [20]. Van de Donk et al. [21] showed that lovastatin negatively affected membrane structure in the myeloma plasma cells and reduced the plasma cell viability, but this was due to the induction of apoptosis and inhibition of proliferation and probably a pleiotropic effect of statins on nuclear receptors in eukaryote cells [22]. Lovastatin interference with nuclear receptors can act synergistically with its ability to inhibit polyisoprenylation and subsequent downstream distortion of intracellular matrix reorganization during cell division [21, 23, 24]. The antiproliferative activity of statins had found increasing use as anticancer drugs in cancer therapy [25, 26]. 

Microbial diversity in the rumen enables ruminants to convert lignocellulosic materials into useful nutrients such as VFA and microbial protein for the host animal. This is complemented by another group of microorganisms, the methanogenic Archaea which coexists within the rumen ecosystem by converting H_2_ and CO_2_ into CH_4_, a greenhouse gas which has been a serious contender for global warming and climate change. Mitigation of rumen CH_4_ production has two advantages: reduction of dietary energy loss, thus improving the efficiency of nutrient utilization by the host animal, and mitigation of enteric CH_4_ production. Both commercial lovastatin and FRSE significantly inhibited CH_4_ production, growth, and cell division of *M. smithii*. Other strategies for mitigation of CH_4_ production in the rumen ecosystem have been extensively researched but with limited success. The idea of applying lovastatin to suppress methanogenesis in methanogenic Archaea [2] has been tested previously. These authors reported that commercial lovastatin inhibited growth of methanogenic Archaea without adversely affecting other cellulolytic bacteria. In practical terms, it is simply uneconomical to use lovastatin as a feed additive for reduction of methanogenesis in ruminants under farm conditions. 

A major difference between Archaea and other microorganisms lies in the structure of their cell membrane. The lipid arm of phospholipids in Archaea is made up of branched isoprenoid, but in other microorganisms, it is fatty acid [27]. The process of isoprenoid and phospholipid biosynthesis in Archaea (Figure 1 in dotted-line markers) share similarities with cholesterol biosynthesis in eukaryotic cells (Figure 1 in solid-line markers) with HMG-CoA reductase as a key enzyme in both pathways, primarily to convert 3-hydroxy-3-3methylglutaryl-CoA (HMG-CoA) to mevalonic acid. Figure 7(a) showed a significant increase in HMG-CoA reductase gene (*hmg*) transcripts in *M. smithii *following lovastatin exposure, a result consistent with increased cellular need for more HMG-CoA reductase enzyme to process a buildup of HMG-CoA because of lovastatin-mediated competitive inactivation of HMG-CoA reductase. It is also evident from Figure 7(a) that H-lovastatin in FRSE was more effective than commercial L-lovastatin in generating a buildup of *hmg *gene transcripts. This is the first report of statins on expression of HMG-CoA reductase gene in Archaea and complements other *in vitro* and *in-vivo* experiments showing increases (seven fold after atorvastatin treatment) and decreases (two-fold after simvastatin treatment) in HMG-CoA reductase gene activation [28] in eukaryote cells. As well, enhancement of the relative expression of *hmg* genes and protein production involved in cholestrol biosynthesis by lovastatin have also been reported for many other systems [28–32]. Essentially, the mode of action of FRSE lovastatin on HMG-CoA reductase in Archaea is similar to its effect in animal cells.

While the experimental evidence so far supports the working hypothesis that interference of the isoprenoid synthetic pathway by lovastatin inhibition of HMG CoA synthetase is primarily responsible for reduced cell growth and decreased methane production, it is likely that the pleiotropic consequences of lovastatin on eukaryote cells in terms of its anti-proliferative and antimetabolic activity may have similar effects on other metabolic pathways in Archaea. For instance, quantitative proteomic analysis has revealed that lovastatin induced perturbation in multiple cellular pathways in HL-60 cells [33]. In the case of Archaea, methane production could also be affected by lovastatin by interference in its synthetic pathway. To assess this possibility, the biosynthetic pathway of methanogenesis is summarized in Figure 2. Methyl H_4_MPT is a pivotal component in this pathway because its synthesis is vital for the production of acetyl-CoA, a key element in cellular processes and metabolism [14]. We selected 3 genes—*hmd*, *adh,* and *fno* that are involved in the synthesis of Methyl H_4_MPT and 3 others responsible for methane synthesis—*mtr, mcr,* and *mta* for real-time PCR assays using the same RNA message transcripts from cultures sampled for *hmg* analysis. The results in Figure 7 show that *hmd* (B), *adh* (C), and *fno* (D) gene transcripts were all depressed in *M. smithii *following exposure to lovastatin. We propose that lovastatin interference of isoprenyl precursor synthesis has caused an increased buildup of acetyl-CoA in the metabolic pool resulting in feedback suppression of genes engaged in the synthesis of Methyl H_4_MPT. Surprisingly, despite a drop in overall CH_4_ production in the cultures, there was an increased expression in the three genes (*mtr, mcr,* and *mta*) responsible for CH_4_ synthesis. We propose that this anomaly is not spurious but represents the transcriptome of *M. smithii* cells that are not dividing because they have been adversely affected by lovastatin. In these cells, *mtr, mcr,* and *mta* gene transcripts (Figures 7(e), 7(f), and 7(g), resp.) are working in cohort to dissipate intracellular pools of Methyl-H_4_MPT. 

In conclusion, we have shown that sufficient levels of biologically active H-lovastatin are produced in rice straws fermented by *A. terreus, *and fermented rice straw extracts are able to disrupt cell wall formation in the chosen rumen test methanogen *M. smithii*. This disruption is associated with decreased CH_4_ production driven in part by interference with isoprenyl synthesis and also through pleiotropic interference of lovastatin in other metabolic pathways. The incorporation of FRSE as a feed additive for ruminants appears to be an economically viable and environmentally sustainable strategy to mitigate CH_4_ production. 

## Figures and Tables

**Table 1:** Primers used in gene expression study.

Official symbol	Descriptor	Primer sequence (5′ → 3′)		Amplification size (bp)
*Met *	16S rRNA	Forward Reverse	GCTCAGTAACACGTGG CGGTGTGTGCAAGGAG	343
*mcrA *	Methyl coenzyme-M reductase, subunit A	Forward Reverse	TTCGGTGGATCDCARAGRGC GBARGTCGWAWCCGTAGAATCC	140
*fno *	F420-dependent NADP reductase	Forward Reverse	GGGTTCAGCAGCAGAAAGG CACATTCAATTGGGTCTGGA	118
*mta *	Methanol : cobalamin methyltransferase	Forward Reverse	ATGTGGTGCAAAAGGACCTC CAGAGTGTGCACAAACAGCA	112
*adh *	Alcohol dehydrogenase	Forward Reverse	AAGAAGTCCCGGAATGTGG TCCGATAGCTCCTTCCCATA	102
*hmd *	Methylene-H_4_MPT dehydrogenase	Forward Reverse	ACCCAGGTGCTGTACCTGAAAT TGTGAATGCAGATCCTCTTGCT	119
*mtr *	Methyl-H_4_MPT : coenzyme M methyltransferase	Forward Reverse	AACAAAGCGGCTTCTGGTGAA CGACACAAGATCCCATTGCAAT	127
*hmg *	HMG-CoA reductase	Forward Reverse	GGCTGTGAATTACCGCATATGG TAACGGTCCGGCTACACCTACA	117
